# Constraint Mechanism of Power Device Design Based on Perovskite Quantum Dots Pumped by an Electron Beam

**DOI:** 10.3390/s22103721

**Published:** 2022-05-13

**Authors:** Yining Mu, Yanzheng Li, Peng Du, Hang Ren, Idelfonso Tafur Monroy, Makram Ibrahim, Guanyu Wen, Dong Liang, Jianshang Feng, Jiayu Ao, Xiangyue Xie, Yumeng Li

**Affiliations:** 1 School of Physics, Changchun University of Science and Technology, Changchun 130022, China; leeyzcust@163.com (Y.L.); dupeng1997@hotmail.com (P.D.); renhang1998@hotmail.com (H.R.); i.tafur.monroy@tue.nl (I.T.M.); makikh@yahoo.com (M.I.); ldcust@163.com (D.L.); s1431049536@163.com (J.F.); adlg123@163.com (J.A.); albery@163.net (X.X.); lifuermosi12@163.com (Y.L.); 2 Chongqing Research Institute, Changchun University of Science and Technology, Chongqing 400020, China; 3 Institute for Photonic Integration, Eindhoven University of Technology, 5600 MB Eindhoven, The Netherlands; 4 Solar and Space Research Department, National Research Institute of Astronomy and Geophysics (NRIAG), Cairo 11421, Egypt; 5 Changchun Observatory, National Astronomical Observatories, Chinese Academy of Sciences, Changchun 130117, China; wengy@cho.ac.cn

**Keywords:** perovskite quantum dots, electron beam pumping, self-saturation luminescence, aging failure

## Abstract

This paper studied the constraint mechanism for power device design based on perovskite quantum dots pumped by an electron beam. Combined with device designing, an experimental system of self-saturation luminescence and aging failure was designed for CsPbBr_3_ films. On this basis, we further completed the self-saturation luminescence and aging failure experiment and constructed a model of self-saturation luminescence and aging failure for CsPbBr_3_ device designing. Three constraints were proposed after analyzing and discussing the experimental data. Firstly, too high of a pumping current density makes it difficult to effectively promote the enhancement of luminescence efficiency. Secondly, radiation decomposition and aging failure of CsPbBr_3_ films are mainly related to the polarized degree of CsPbBr_3_ nanocrystals. Thirdly, by increasing the pumping electric field, the pumping energy can be effectively and widely delivered to the three-dimensional quantum dots film layer space, and there is a nonlinear relationship between the attenuation of the pumping energy density and the increment of the pumping electric field, which will effectively avoid the local high-energy density of instantaneous optical pumping.

## 1. Introduction

In recent years, all-inorganic cesium lead halide perovskite materials CsPbX_3_ (X = I, Br, Cl) have attracted a lot of attention in academia due to their excellent optical properties [1,2,3,4]. For example, the emitting wavelength of CsPbX_3_ nanocrystals can be effectively affected by adjusting the composition of halogen elements (Cl/Br and Br/I ratio) [5,6,7,8,9,10,11]; this approach is often used in bulk semiconductors (such as AlGaAs) or nanocrystals [12]. In particular, there are few defects in the metal halide perovskite material since it has almost no inter-band defect state, which causes its luminescent quantum yield rates as high as 90% [13,14]. In 2018, Lin et al. reported that the external quantum efficiency of perovskite LED had reached 20%, which is close to the performance of the best organic LED at present [15]. In addition, the carrier recombination efficiency and fluorescence lifetime of the CsPbX_3_ material can be obviously and effectively improved by taking advantage of the resonance energy transfer property between noble metal plasmon and perovskite luminescent materials [16,17]. Therefore, importing noble metal nanocrystals into CsPbX_3_ light-emitting devices could be an effective method for achieving fluorescence enhancement and improving the quantum efficiency and frequency response of devices obviously [18,19]. However, in terms of device designing, there is a power gain bandwidth product constraint on the current injection quantum dot LED. Theoretically, in order to satisfy high-power device designing, widening the cross-sectional area of the PN junction and improving the pumping current’s intensity in the PN junction is the most effective technical method; however, this method will directly lead to an increase in the PN junction capacitance and further disturb or restrict the advantages of quantum dot LED in terms of low fluorescence lifetime. For this constraint, a number of novel pumping models and power-type device structures have been proposed in academia. Therein, the most outstanding achievement is the first report of a CsPbX_3_ nanocrystal random laser based on ultrafast optical pumping in 2015, whose optical gain was as high as 450 cm^−1^ [11]. In current academia, regarding the enhancement of random lasing efficiency and the quality factor of CsPbBr_3_ pumped by optics, the main methods include lowering the wavelength of pumping laser [20,21,22,23], shortening the pumping time gap [24,25,26,27], manufacturing a complex noncrystalline structure [28,29,30,31] and so on. Among them, the most typical method is enhancing the surface roughness of CsPbBr_3_ films [32,33,34,35,36], which can cheaply increase the probability of CsPbBr_3_ nanocrystalline pumped by multiple photons at the same time. However, whether it is to compress the pump time slot or to increase the surface roughness, it is to increase the lasing probability by increasing the pump energy density of nanocrystals, which will undoubtedly lead to an enhancement in the unnecessary local thermal density and thermal effect interference. Therefore, improving the efficiency of the pumping model is an effective means for power-type device design.

Targeting this bottleneck, in 2020, our team firstly reported random lasing perovskite quantum dot films pumped by an electron beam (EB) and proved that, compared with optical pumping, there are some great advantages in terms of energy conversion efficiency and power output characteristics to the EB-pumping model [37]. However, luminescence self-saturation, quantum dot materials’ aging failure and radiation decomposition caused by high pumping current injection will immediately restrict the power properties of the device design. For this purpose, this paper carried out power-type luminescence experimental research based on perovskite quantum dot devices pumped by an EB and reported its self-saturation luminescence and aging failure mechanism, and we further revealed some important constraints on the design of power-type devices according to the above experimental results.

## 2. Materials and Methods

### 2.1. CsPbBr_3_ Solution Preparation and Luminescence Film Characterization

CsPbBr_3_ quantum dots were synthesized in an oil phase protected by an inert gas using cesium oleate and lead bromide as precursors through thermal injection and rapid ion exchange. After centrifugation and purification by high-speed centrifuge, the CsPbBr_3_ quantum dot solution of all-inorganic metals with hexane as the solvent and a concentration of 10 mg/mL emitting green fluorescence was prepared. The conductive glass (indium tin oxide (ITO)) substrate was ultrasonically cleaned with acetone, absolute ethanol and deionized water for 15 min, dried by a nitrogen gun and placed in a drying oven at 80 °C for 30 min. The prepared quantum dot solution was uniformly stirred by a magnetic stirrer to obtain a yellow transparent CsPbBr_3_ quantum dot spin coating solution. The prepared quantum dot solution was uniformly stirred by a magnetic stirrer to obtain a yellow transparent CsPbBr_3_ quantum dot spin coating solution. Then, the glue machine was used. The quantum dot spin coating solution obtained by the one-step spin coating method is dropped on a conductive glass substrate through a special syringe, where the homogenizing speed of 900 r/min is maintained for 5~10 s before dripping, and the homogenizing speed of 3500 r/min is maintained after dripping 10~20 s. Finally, spin coating resulted in uniformly translucent CsPbBr_3_ quantum dot films. The number of syringe droplets was determined by means of a microelectronic analytical balance with a reading accuracy of 0.01 mg. The experiment shows that under certain conditions, the higher the spin coating speed, the longer the gelling time, and the thinner the quantum dot films.

**Figure 1:**
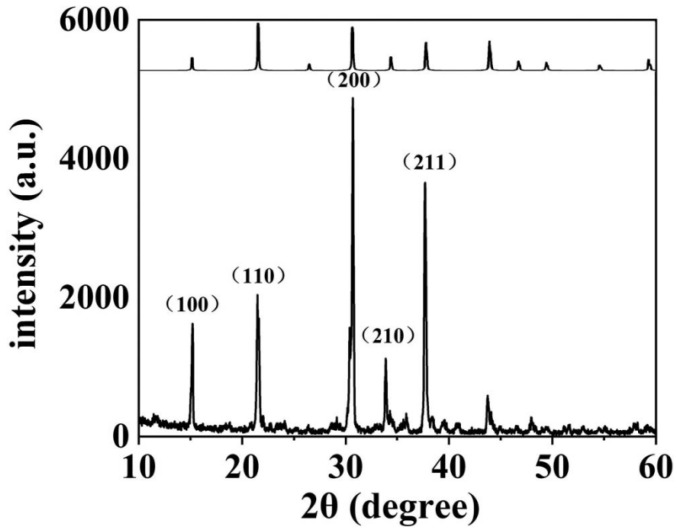
X-ray diffraction patterns of CsPbBr_3_ nanocrystals.

**Figure 2:**
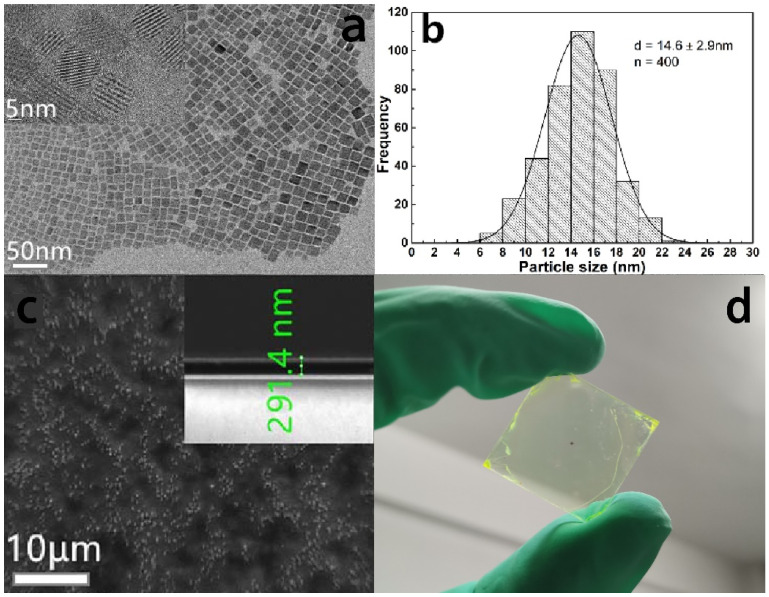
(**a**) TEM characterization of CsPbBr_3_ nanocrystals. (**b**) The size distribution of the nanocrystals. (**c**) Scanning electron microscopy (SEM) characterization of CsPbBr_3_ perovskite thin films with thicknesses of 300 nm. (**d**) Macroscopic films.

The X-ray diffraction pattern of CsPbBr_3_ quantum dots is shown in Figure 1. The characteristic peaks are 15.1°, 21.5°, 30.4°, 34.2° and 37.6°, respectively, corresponding to crystal structures {001}, {110}, {002}, {210} and {211}, which is consistent with the standard diffraction pattern obtained from the Joint Committee on Powder Diffraction Standards (JCPDS) database (No. 01-072-7929), and have a cubic crystal structure. In addition, we characterized the CsPbBr_3_ nanocrystals by transmission electron microscopy (TEM). As shown in Figure 2a, the CsPbBr_3_ nanocrystalline morphology effect used herein is shown. Figure 2b shows the size distribution of the nanocrystals, which shows that the average size of the nanocrystals is about 15 nm. Figure 2c shows the scanning electron microscope images of CsPbBr_3_ quantum dot films. Additionally, Figure 2d is the macroscopic feature of final luminescence films. As can be seen in Figure 1 and Figure 2, the experimental solution belongs to normal CsPbBr_3_ films, which can be easily replicated in experimental results for other scholars.

### 2.2. Measurement Methods of Space–Time Coherence for Self-Saturation Luminescence Experiment

**Figure 3:**
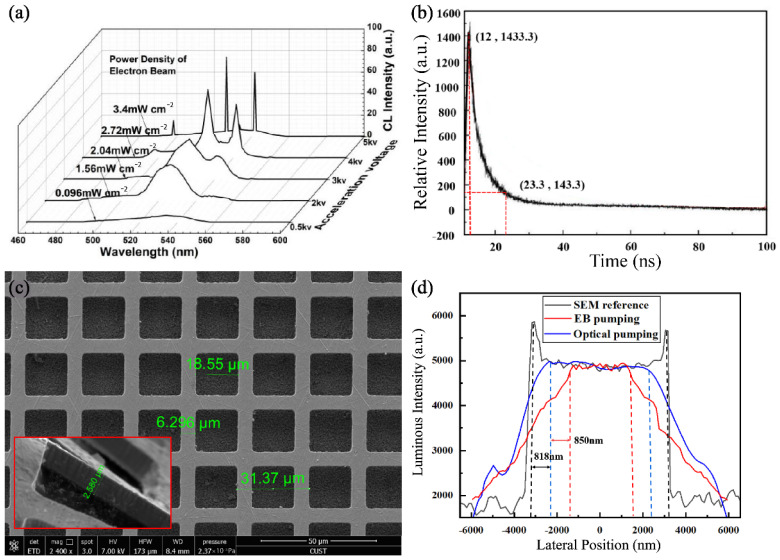
(**a**) EB-pumping spectra of CsPbBr_3_ quantum dot thin films by different acceleration voltages. (**b**) Transient luminescence of the CsPbBr_3_ films. (**c**) Space size of the metal film net. (**d**) Spatial coherence comparison of both luminescence models.

The radiation spectrum changes in CsPbBr_3_ quantum dot films bombarded with an accelerated EB are shown in Figure 3a. In order to make CsPbBr_3_ nanocrystals continuously pumped by multiple scattered electrons before depolarization, accurately measuring the temporal coherence and spatial coherence of perovskite quantum dots pumped by an EB will be crucial for the subsequent design of self-saturation luminescence experiments. For this purpose, our team completed a transient luminescence experiment on CsPbBr_3_ films in 2020 [38]. There is no denying that the testing result in Figure 3b is similar to some reports in academia [39,40]. The depolarization delay of CsPbBr_3_ films is only around several ns, even though there is a wide band gap. In particular, the temporal coherence of CsPbBr_3_ luminescence pumped by an EB should be in the order of 10 ns. Moreover, the spatial coherence of EB scattering in the CsPbBr_3_ films has been estimated in the following paper. In the vacuum testing system, the CsPbBr_3_ films are located at the focal plane of the microscope with a 40-times magnification. The sampling rate and accuracy of the CMOS sensor is around 140 nm, which is greatly superior to the optical transfer ability of the microscope in the 550 nm band. A metal film net is placed on the surface of CsPbBr_3_ films, which can block pumping EB to shape the spatial frequency information for CsPbBr_3_ luminescence. By observing the attenuation characteristics of space frequency information, the spatial coherence of EB scattering has been basically confirmed. According to the above methods, the characterization of the metal film-net can be shown in Figure 3c. The space frequency spectrum of EB pumping and optics pumping and SEM were measured with a pumping voltage of 5kV and are shown in Figure 3d. As shown in the figure, there is EB scattering of 800 nm in the CsPbBr_3_ films. The spatial coherence of EB scattering should be around the submicron order.

### 2.3. Measurement Methods of CsPbBr_3_ Self-Saturation Luminescence

**Figure 4:**
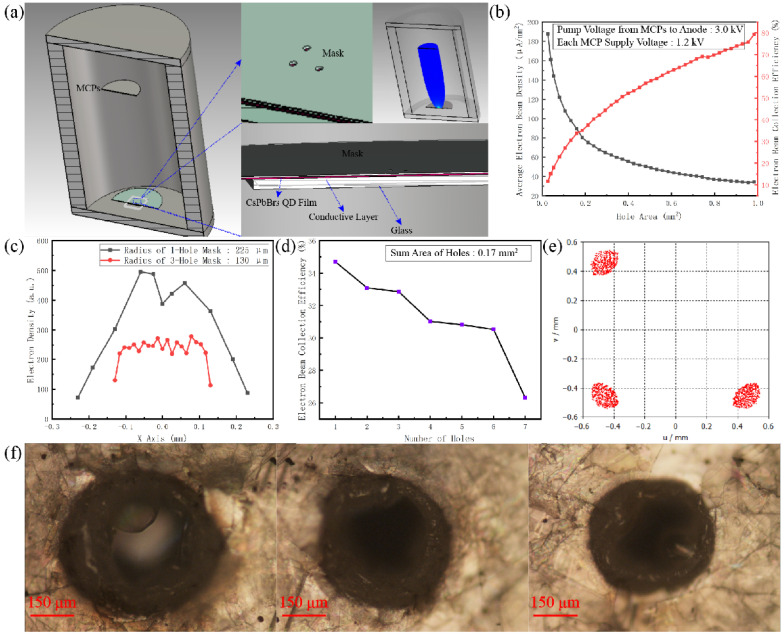
(**a**) Design and simulation of electron optical focusing system. (**b**) The effect of mask hole area on EB collection efficiency and focusing gain pump power at MCPs output of 40 μA. (**c**) The effect of mask hole radius on EB focusing distribution. (**d**) Relationship between the number of hole masks and EB collection efficiency. (**e**) Lectronic focal spot shapes. (**f**) Characterization of the three-hole anodes.

According to the spatial and temporal coherence experimental results of the CsPbBr_3_ films pumped by EB, only when the pumping current’s density reaches the order of magnitude of μA/mm^2^ will the CsPbBr_3_ nanocrystals probably be bombarded by two or multiple electrons in time and space. In other words, at this time, the pumping energies of multiple electrons overlap with each other in the time–space domain for the same nanocrystal. Therefore, designing and constructing a high-quality EB-pumping source with a current density of several dozen μA/mm^2^ will be very important for the subsequent self-saturation luminescence experiment. In a traditional cathode-luminescence (CL) system, although the thermionic cathode has a strong ability to output electrons, it will emit electrons accompanied by strong background radiation. Additionally, the background radiation overlaps with the self-saturation luminescence spectrum to be detected, which will directly disturb and affect the final measurement accuracy of self-saturation luminescence. Based on this, in this paper, an ideal electron source is yielded by a Au cathode triggered by UV, and the power of the electron source is multiplied to shape the EB by one or two microchannel plates (MCPs). The scheme in this paper can avoid the mutual interference between the triggering light source and the self-saturation luminescence spectrum to be measured. However, the self-saturation output currents of MCP had been limited by its plate currents, and extreme output currents of the EB-pumping source based on MCPs are very difficult to exceed the order of tens of μA/mm^2^. Therefore, it is very important for the final self-saturation luminescence detection to focus on the output energy density of the MCPs through the electron optical system. To this end, we took advantage of the CST (three-dimensional electromagnetic field simulation software) platform to optimize the design and system simulation for the EB focus system, and the specific design and simulation results are shown in Figure 4.

**Figure 5:**
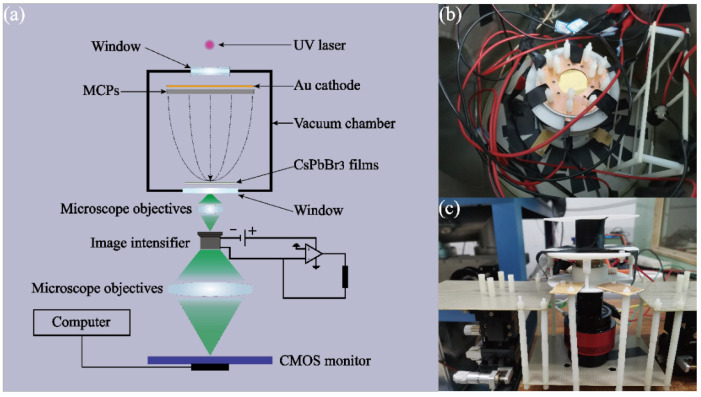
(**a**) Self-saturation luminescence experimental system. (**b**) Vacuum device part. (**c**) Luminescence testing system.

In the simulation model of Figure 4a, focusing the EB more effectively and collecting more of the pumping power’s surface density need to be further considered. According to the model in Figure 4a and conditions of Figure 4b, the output currents of MCPs can reach around 40 μA. When the mask hole on the anode is reduced to below 1 mm^2^, the collection efficiency of focusing the EB will decline rapidly, and the surface density of pumping power focused into the hole mask will also be enhanced promptly. From the simulated data in Figure 4b, there is an optimal parameter for the whole EB-focusing system. In the EB-focusing system, when the area of the hole mask is around 0.17 mm^2^, the collection efficiency and pumping-power focusing will be balanced effectively. In addition, according to the further simulation of the inside of the hole mask, if the diameter of the hole mask is too big, the distribution of EB focusing, as shown in Figure 4c, will be like a Gaussian distribution. Namely, an excessive diameter makes it difficult to smooth the distribution of EB focusing, whereas taking advantage of more tiny holes to assemble a hole of 0.17 mm^2^ will avoid an unsmooth distribution. Of course, Figure 4d also shows the relationship between the number of hole masks and the EB collection efficiency. Based on the above analysis, in the following experiment, we selected three hole masks to focus the EB. The whole area of three hole masks is around 0.17 mm^2^. Their simulated distribution is shown in Figure 4e. In order to achieve the electron focusing effect in Figure 4e, we took advantage of a ps laser to carve three holes with a diameter of 260 μm on a mica sheet with a thickness of 0.1 mm, whose characterizations are shown in Figure 4f by a microscope. The mica sheet with three holes was placed on the CsPbBr_3_ luminescence films to shape focus anodes. The schematic diagram of the self-saturated luminescence experimental device is shown in Figure 5a. Figure 5a mainly includes the vacuum device part and the luminescence testing part. In the vacuum device part, a Au photocathode can be triggered by the UV laser to yield some ideal photoelectrons. The photoelectrons are amplified into EB by the MCPs. By the EB-focusing system and external pumping field, the EB is charged enough kinetic energy to bombard the CsPbBr_3_ films across the three holes. In the luminescence testing system, as the anode of the vacuum device part, the three holes and CsPbBr_3_ films are placed on the focus plane of the microscope in the objective space. The light from the three holes will be transferred to the photocathode of the image intensifier located at the focus plane of the microscope in the imaging space. The intensity variation in CsPbBr_3_ films’ self-saturation luminescence can be quantificationally measured from anode currents of the image intensifier by remoting voltage between both sides of MCPs. The imaging information of the three holes can be also collected by the below CMOS monitor. The imaging information can qualitatively and immediately reflect the degree of self-saturation luminescence. The vacuum device part and luminescence testing system have been shown in Figure 5b,c, respectively.

### 2.4. Measurement Methods of CsPbBr_3_ Luminescence Aging Failure

**Figure 6:**
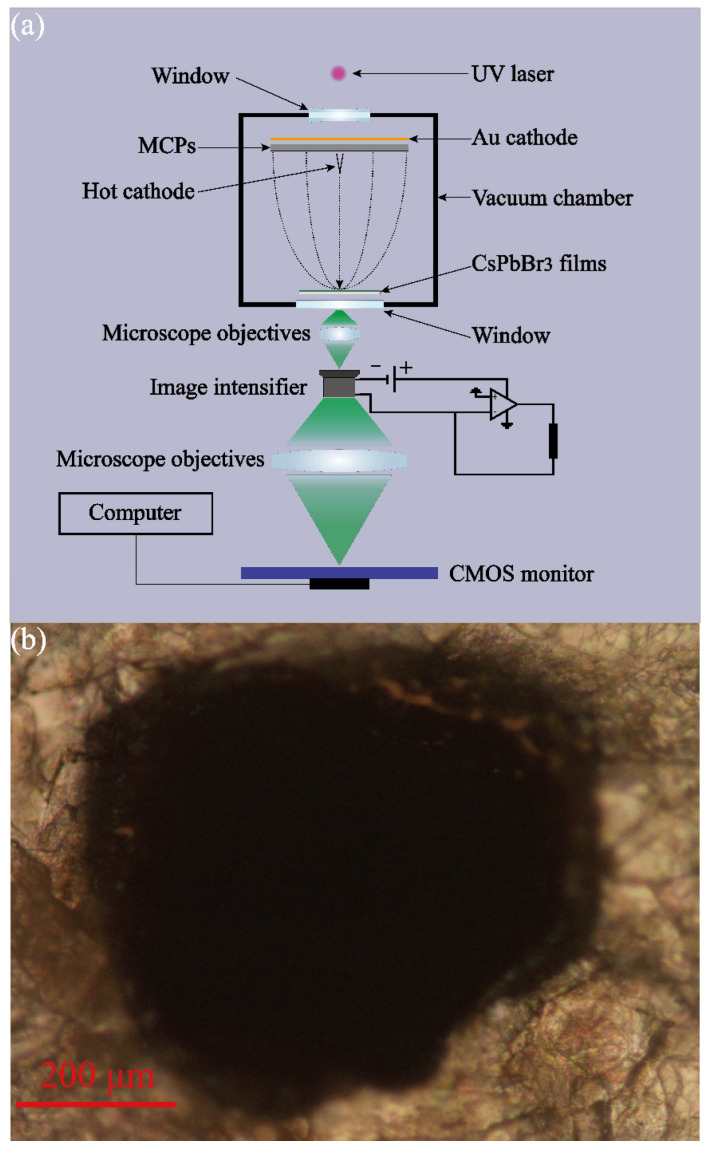
(**a**) Aging failure experimental system. (**b**) Characterization of the square anodes.

The main difference between luminescence aging failure and self-saturation luminescence testing lies in the high-power pumping duration. The luminescence aging failure experiment needs the EB-pumping system to work continuously and stably for more than one hour in the high-power mode. However, because of the obvious multiplier fatigue property after working long, MCPs are not suitable for aging failure testing. Therefore, in the subsequent aging failure experiments, we took advantage of a dual-mode EB source to pump the CsPbBr_3_ films in turns. In the first step, the CsPbBr_3_ films are constantly pumped and aged by a tungsten-thorium alloy direct thermal cathode whose currents output can reach 10 μA steadily. Every few minutes, the variation in the luminescence property can be sampled by the MCP pumping system. In addition, in order to reduce testing errors, the three holes on the mica sheet are enlarged into a square of 0.5 mm × 0.5 mm. The aging failure experiment and characterization of the square on the mica sheet are shown in Figure 6.

## 3. Results and Discussion

### 3.1. CsPbBr_3_ self-Saturation Luminescence and Aging Failure Experimental Results

**Figure 7:**
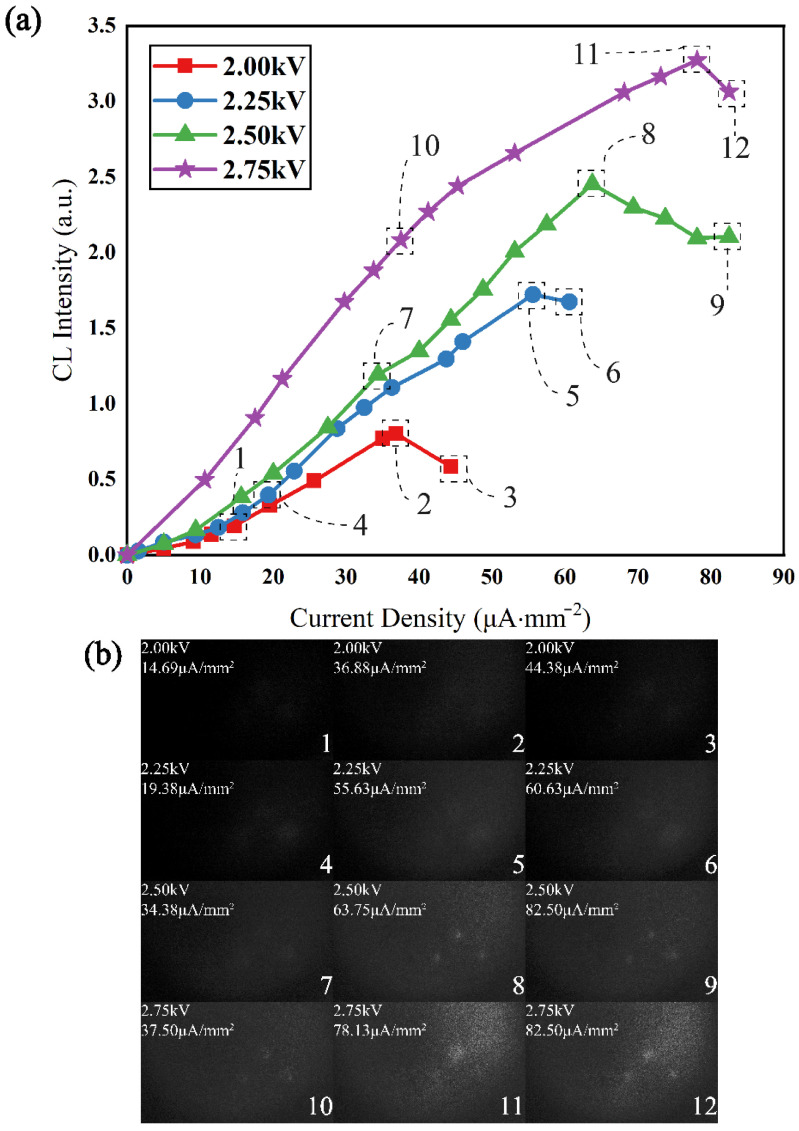
(**a**) CsPbBr_3_ self-saturation luminescence experimental trend. (**b**) CsPbBr_3_ self-saturation luminescence process and results.

Based on the system and devices shown in Figure 5a, we carried out the following experimental steps. Firstly, by accurately adjusting the voltage between both sides of the MCPs from 1200 V to 600 V, the current’s intensity of pumping EB is accurately decreased from 10 μA to 0.1 μA, and further, the pumping current’s density across the three holes can be effectively operated. The anode current’s intensity of the image intensifier is closely related to the luminescence intensity from the three holes only. Therefore, a self-saturation luminescence of CsPbBr_3_ pumped by an EB can be directly modeled in Figure 7a. As can be seen, when selecting pumping voltages, CsPbBr_3_ luminescence will enter into the self-saturation region to varying degrees. In addition, in order to easily and directly reflect the physical phenomena and process of CsPbBr_3_ self-saturation luminescence, we took advantage of the image intensifier and the CMOS monitor to capture all of the self-saturation steps of luminescence. Of course, the imaging picture from the CMOS monitor will be affected by the image intensifier’s anode voltage. However, errors from imaging gain can be tolerated. The whole self-saturation luminescence process is shown in Figure 7b, and Figure 7b mainly shows key data from Figure 7a. Therein, the data of Figure 7a are from anode currents of the image intensifier in Figure 5a. Additionally, detailed values can be collected by the operational amplifier of Figure 5a. The information in Figure 7b was mainly captured by the CMOS monitor. Therefore, Figure 7a shows the process of self-saturation luminescence quantificationally, and Figure 7b reflects the results of self-saturation luminescence qualitatively. Figure 7a is closely related to Figure 7b.

**Figure 8:**
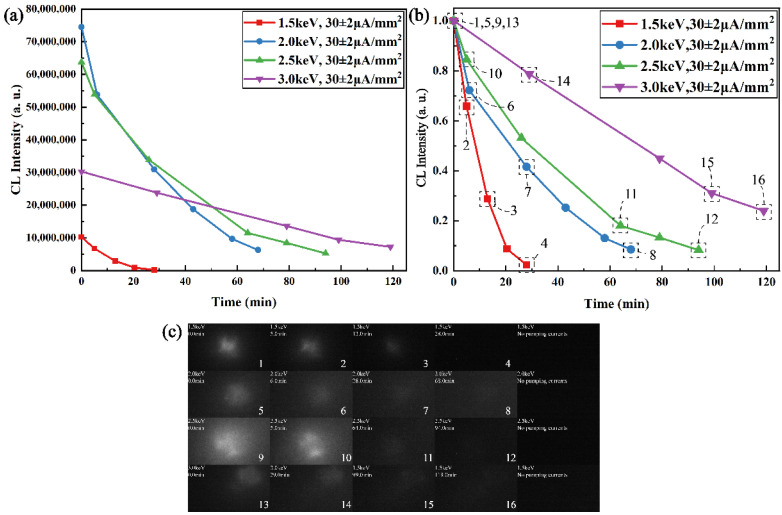
(**a**) Experimental trend of non-normalized CsPbBr_3_ luminescence aging failure. (**b**) CsPbBr_3_ luminescence aging failure experimental trend. (**c**) CsPbBr_3_ luminescence failure aging process and results.

For another aging failure experiment, compared with the system and devices in Figure 6a, we made similar testing steps to acquire aging failure data of CsPbBr_3_ luminescence alternately. Different from the self-saturation luminescence experiment, the CsPbBr_3_ quantum dot films need to be replaced after each luminescence aging failure experiment. Therefore, the results of the luminescence aging failure experiment cannot be used to compare the luminescence intensity among experimental results with different pumping voltages in Figure 8c. Because there were different quantum dot films in the aging failure experiment and monitor system in Figure 6a, Figure 8a only shows the aging failure trend of the different films. In order to find the rule more clearly from the experimental results of luminescent aging failure, we normalized the experimental results. The aging failure experimental trend and process of CsPbBr_3_ luminescence are shown in Figure 8a–c, respectively, and the expression model of Figure 8 is similar to that of Figure 7.

### 3.2. Discussion and Analysis

**Figure 9:**
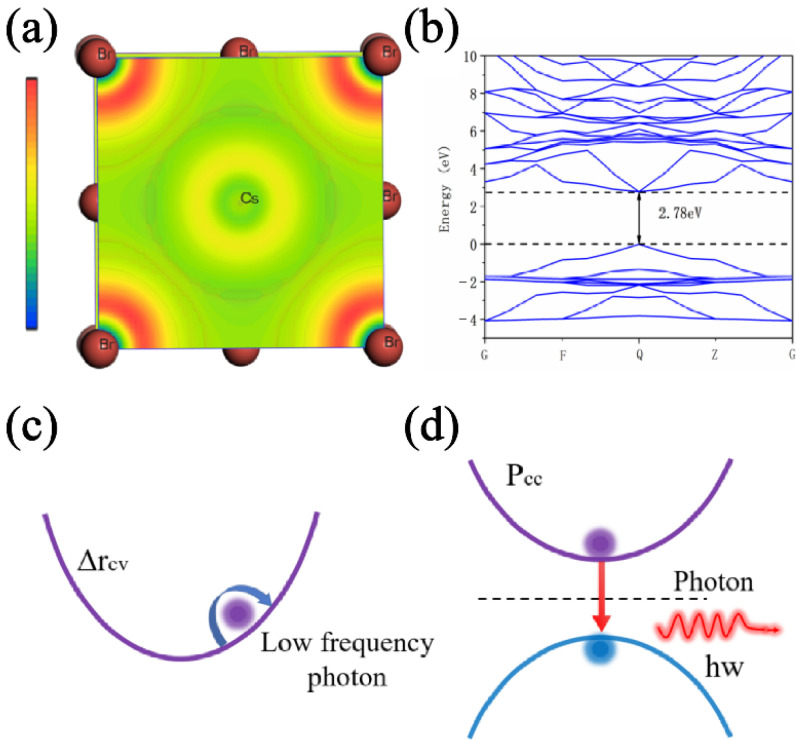
(**a**) Electron localization function of CsPbBr_3_ at (001) plane. (**b**) Band structure of the CsPbBr_3_. (**c**) Low-frequency photon radiation model. (**d**) Visible photon radiation model.

From the micro aspect, CsPbBr_3_ Perovskite quantum dots (PQDs) bulk material belongs to a typical centrosymmetric structure. However, a number of actual factors will disturb the inversion symmetry [41], such as the Pb^2+^ lone pair, sample defects, and surface effects. Therefore, the lone pair of Pb atoms will also be activated and has a weak displacement in CsPbBr_3_ pumped by an ultrafast excitation source. Figure 9a shows the electron localization function of CsPbBr_3_ on the (001) plane. The asymmetry of the electron density around the Pb atom shows that the cation displacement will disturb the local symmetry, which will yield a high-order shift current in CsPbBr_3_. As we know, CsPbBr_3_ belongs to a typical direct bandgap material structure. Its band structure is shown in Figure 9b, and its valence band is mainly attributed to the Pb s-orbitals and Br p-orbitals, while its conduction band is mainly attributed to the Pb p-orbitals. The lattice of CsPbBr_3_ is distorted with the mass center of the metal cations shifted relative to the oxygen anions, given the fact that the valence band has a larger contribution from Br p-orbitals, while the conduction band contains a larger contribution from Pb s and p-orbitals. Therefore, the electron density in the valence band is mainly distributed around the atoms, while the electron density in the conduction band is relatively shifted between atoms, which will lead to a relative center-of-charge shift between the conduction band and the valence band [42]. The displacement vector is Δr_cv_ = r_c_ − r_v_, where r_v_ is the charge center before electron activation and r_c_ is the charge center after electron activation. The depolarization process within CsPbBr_3_ can be shown in Figure 9c,d. Once a lattice of CsPbBr_3_ was polarized by a high-energy electron, a (virtual) electron and a (virtual) hole appear in the conduction band and valence band, respectively. The electron also has momentum p_cc_ = ћkm_0_/m_c_ in the conduction band with the wave vector k, where ћ is the reduced Planck constant, m_0_ is the free mass of the electron and m_C_ is the effective mass of the electron at the base of the conduction band. Low-frequency photon radiation is generated by the “shift” dipole moment, as shown in Figure 9c. Meanwhile, a visible photon is emitted as the electron and hole recombine and the energy is once again conserved, as shown in Figure 9d. However, when the pumping current’s density increases, the probability that one CsPbBr_3_ nanocrystalline is pumped and bombarded simultaneously by multiple electrons with upper kinetic energy is also enhanced. The increment of the probability may raise the conversion efficiency of low-frequency photon radiation. However, theoretically, the emitting efficiency of a visible photon is hard to improve. On the contrary, too high of a pumping current density can directly yield a local thermal effect and reduce the luminescence effect, which was proven by the self-saturation luminescence data in Figure 7. Moreover, too high of a pumping current density will also cause a large number of CsPbBr_3_ nanocrystalline to be extremely polarized. The electron of the valence band is excited deep into the conduction band, which may increase the probability of the “shift” dipole moment being unrecoverable. In other words, the “shift” dipole moment cannot recover, which causes the radiation decomposition and luminescence aging of the CsPbBr_3_ nanocrystalline. The process of radiation decomposition and luminescence aging is proven by the data in Figure 8.

From the macro aspect, from comparing the mass of electrons to photons, EB pumping cannot only polarize nanocrystals located at the surface of CsPbBr_3_ films like optical pumping but also randomly scatter in the films and polarize a large number of CsPbBr_3_ nanocrystals in the inner of films. Therefore, a higher pumping voltage makes the pumping energy spread wider layers of CsPbBr_3_ films, which effectively reduces the volume density of the pumping energy, further postpones the threshold of self-saturation luminescence and prolongs the lifetime of CsPbBr_3_ films under a high-current injection model. This conclusion has also been proven in Figure 7 and Figure 8.

## 4. Conclusions

According to the above experiments, simulation analysis and theoretical modeling, this paper obtains the following three constraints for power device design based on perovskite quantum dots pumped by EB. Firstly, too high of a pumping current density increases the probability that a nanocrystal will be pumped by multiple electrons at the same time, so most of the energy is used to pump electrons to the deep energy level, which makes it difficult to effectively promote the enhancement of the luminescence efficiency. Additionally, self-saturation of the pumping energy’s density will also cause unnecessary thermal effects, which will further limit the luminescence efficiency. Secondly, excessive polarization of CsPbBr_3_ nanocrystals will lead to radiation decomposition and aging failure of CsPbBr_3_ films. How to accurately and effectively avoid the excessive polarization of quantum dot nanocrystals is of great significance for subsequent device design. Finally, compared with the optical pumping model, the EB-pumping model has obvious advantages in terms of power device design. The pumping energy can be effectively transferred to a wider three-dimensional quantum dot film layer space with the increase in the pumping electric field, and there is a nonlinear relationship between the increment of the pumping electric field and the attenuation of the pumping energy density, which can effectively avoid the local high energy density caused by instantaneous pumping and thus reduce the unnecessary local thermal effect. To sum up, a perovskite quantum dot device pumped by an EB has outstanding research potential in the follow-up new transient light source and high-power terahertz radiation source.

## Data Availability

Not applicable.
